# Relationships of familial sexual stigma and family support with internalized homonegativity among lesbian, gay and bisexual individuals: The mediating effect of self-identity disturbance and moderating effect of gender

**DOI:** 10.1186/s12889-022-13815-4

**Published:** 2022-08-01

**Authors:** Chung-Ying Lin, Mark D. Griffiths, Amir H. Pakpour, Ching-Shu Tsai, Cheng-Fang Yen

**Affiliations:** 1 Institute of Allied Health Sciences, College of Medicine, National Cheng Kung University, Tainan, Taiwan; 2 Department of Occupational Therapy, College of Medicine, National Cheng Kung University, Tainan, Taiwan; 3 Department of Public Health, College of Medicine, National Cheng Kung University, Tainan, Taiwan; 4 Biostatistics Consulting Center, National Cheng Kung University Hospital, College of Medicine, National Cheng Kung University, Tainan, Taiwan; 5 International Gaming Research Unit, Psychology Department, Nottingham Trent University, Nottingham, UK; 6 Department of Nursing, School of Health and Welfare, Jönköping University, Jönköping, Sweden; 7 Department of Child and Adolescent Psychiatry, Chang Gung Memorial Hospital, Kaohsiung Medical Center, Kaohsiung and School of Medicine, Chang Gung University, Taoyuan, Taiwan; 8 Department of Child and Adolescent Psychiatry, Chang Gung Memorial Hospital, Kaohsiung Medical Center, 32 Dapi Rd. Niaosong Dist., Kaohsiung, 83341 Taiwan; 9 Department of Psychiatry, Kaohsiung Medical University Hospital, and School of Medicine College of Medicine, Kaohsiung Medical University, 100 Tzyou 1st Road, Kaohsiung, 80708 Taiwan; 10 College of Professional Studies, National Pingtung University of Science and Technology, Pingtung, Taiwan

**Keywords:** Family, Self-identity disturbance, Internalized homonegativity, Sexual minority, Psychological wellbeing

## Abstract

**Background:**

The mediators of the association between familial attitudes toward sexual orientation and internalized homonegativity among lesbian, gay, and bisexual (LGB) individuals have not been well examined.

**Methods:**

A cross-sectional survey study was carried out to examine the (i) associations of familial sexual stigma and family support with internalized homonegativity among young adult LGB individuals in Taiwan, and (ii) mediating effect of self-identity disturbance and the moderating effect of gender. Self-identified LGB individuals (*N* = 1000; 50% males and 50% females; mean age = 24.6 years) participated in the study. Familial sexual stigma, family support, self-identity disturbance, and internalized homonegativity were assessed. Structural equation modeling was used to examine relationships between the variables.

**Results:**

The results indicated that familial sexual stigma was directly associated with increased internalized homonegativity, and indirectly associated with increased internalized homonegativity via the mediation of self-identity disturbance among LGB individuals. Family support was indirectly associated with decreased internalized homonegativity via the mediation of low self-identity disturbance. The direct association between family support and internalized homonegativity was only found among lesbian and bisexual women but not among gay and bisexual men.

**Conclusions:**

Program interventions for familial sexual stigma, family support, and self-identity disturbance are warranted to help reduce internalized homonegativity among LGB individuals.

## Background

### Internalized homonegativity among lesbian, gay, and bisexual individuals

Lesbian, gay, and bisexual (LGB) individuals often experience public stigma due to their sexual orientation such as bullying, hate crimes, and structural stigma derived from heterosexism [1]. LGB individuals may endorse the public stigma due to their sexual orientation and develop internalized homonegativity [2]. According to the minority stress theory [2], public stigma rooted in heterosexism and subsequent internalized homonegativity belong to distal and proximal stressors, respectively; both contribute the development of mental health problems among LGB individuals. Expanding upon Meyer’s model [2], Hatzenbuehler [3] hypothesized that internalized homonegativity may mediate the relationship between public stigma and mental health problems.

Research has demonstrated that internalized homonegativity is a multifactorial construct [4–6]. For example, according to the Measure of Internalized Sexual Stigma for Lesbians and Gay Men (MISS-LG) [7], internalized homonegativity comprises three fundamental dimensions: “identity (an enduring propensity to have a negative self-attitude as sexual minority and to consider sexual stigma as a part of a value system and identity), social discomfort (the fear of public identification as a lesbian or gay man in the social context, and disclosure in private and professional life), and sexuality (the pessimistic evaluation of intimate gay or lesbian relationships’ quality and duration and a negative conception of gay or lesbian sexual behaviors)” [7]. Research has shown that internalized homonegativity may endanger LGB individuals’ mental health [8–12] and social relationships [13], increase sexual behaviors increasing the risk of contracting HIV [12, 14, 15], and decrease the intention to access medical care services [16]. Consequently, internalized homonegativity is an important health issue and warrants prevention and intervention among LGB individuals. Contrarily, positive identity such as high self-awareness, authenticity, belonging to the LGB community, intimacy, and perceived social justice assessed by the Lesbian, Gay, and Bisexual Positive Identity Measure contribute to psychological well-being [17–19]. Preventing internalized homonegativity and enhancing positive identity among LGB individuals are therefore needed. Examining the factors that influence the formation of internalized homonegativity is the essential step to develop intervention programs. Research has found that an older age of identification of sexual orientation [20], being religiously active [21], having more lifetime heterosexual attractions [21], being more interested in having children and a child-centered family life [21] are significantly associated with higher internalized homonegativity among LGB individuals.

### Role of familial sexual stigma among internalized homonegativity among LGB individuals during early adulthood

According to the socio-ecological theory [22], internalized homonegativity is the result of the interaction between the individuals and their environments (e.g., microsystem, mesosystem, exosystem and macrosystem). Family is one of the microsystems in which the individuals are embedded; therefore, familial contexts may contribute to the formation and maintenance of self-identification. Health professionals have recognized the importance of family environment for the health of LGB individuals and recommended that healthcare providers educate parents about the health impact of familial support [23, 24]. Familial sexual stigma indicates the ignorance, prejudice and discrimination enacted by family members toward sexual minorities [25–27]. Familial sexual stigma may manifest through a variety of negative attitudes and behaviors, including keeping silent about sexual orientation, sexual orientation-related rejection, bullying, and harassment [25–27]. Research has shown that familial sexual stigma not only contributes to negative health outcomes [25, 26] but also hones the development of internalized homonegativity among LGB individuals [28, 29]. LGB individuals may internalize the moral condemnation regarding sexual minorities they heard from their own family members and conceal their identity from their families of origin to avoid experiences of stigma [29]. Families’ negative reactions to coming out may also exacerbate LGB individuals’ internalized homonegativity [28]. Although there is a direct association between familial sexual stigma and internalized homonegativity, other factors that mediate this association have not been examined.

### Role of family support in internalized homonegativity among LGB individuals during early adulthood

Research has shown that low family support is associated with suicidality, distress, depression, hopelessness, and substance use among LGB individuals [23, 30–32]. Family support also buffers the mediating effect of emotional symptoms in the association between homophobic bullying and sedative/hypnotic use among gay and bisexual men [33]. However, the findings of previous studies examining the association between family support and internalized homonegativity among LGB individuals have been mixed. A cross-sectional study in Israel reported that family support had a positive impact on self-acceptance of sexual orientation among adolescent and young adult LGB individuals [34], whereas a two-year prospective study in the United States reported family support did not predict the level of internalized homonegativity among adolescents and young adult men who have sex with men [35]. Consequently, the mediators of the association between family support and internalized homonegativity among LGB individuals warrant further study.

### Mediating effect of self-identity disturbance

According to the psychosocial developmental theory [36], people explore any opportunities and options available to them and start to make commitments to people around; they undertake roles that they define for themselves and then develop normative self-identity during the process. Self-identity formation is one of the developmental tasks in adolescence and matures in young adulthood. A matured self-identity includes the acceptance of physical changes, development of social and emotional competencies and self-efficacy, and the balance between autonomy and interdependence [37]. People who attain the full process of personality development and consolidate their self-identity can own consistent beliefs and values across time and contexts, indicating having established independent and emancipated self-identity [38]. However, people may fail in developing a normative identity and have a confused self-identity. There are several types of self-identity confusion identified in previous studies [39–41]. People who lack the inability to commit to typical roles and tend to adopt the values, attitudes, beliefs, thoughts, feelings, and problems of others in adulthood may have the disturbed identity [39]. People who fail to make commitments to others, undertake roles that they define for themselves, and express consistent beliefs and values across time and contexts may have the unconsolidated identity [40]. People who shift their self-image suddenly and dramatically with respect to aspirations for future goals and vocation, sexual orientation identity, and types of friends may have the lack of identity [41].

In addition to developing self-identity, establishing sexual orientation is also one of important developmental tasks during adolescence [38]. LGB individuals usually become aware of same-sex attractions, questioning one’s sexual orientation, self-identifying as LGB, coming out to others, engaging in sexual activity, and initiating a romantic relationship between the period of early adolescence and early adulthood [42]. According to the socio-ecological theory [22], individual factors, environmental factors, and the interaction between individuals and environments influence the formation of self-identity and sexual orientation. Regarding individual factors, self-identity and sexual orientation may have an effect on one another in the process of exploration [43]. Regarding the environmental factors, various severities of pressure from outside sources may result in different impacts on the development of self-identity. Helson and Roberts [44] reported that experiencing an optimal level of challenge is critical for people to develop mature ego, whereas Anthis [45] found stressful life events may aggravate the burden of exploring self-identity and decrease the stableness of identity and commitments. Research has also reported that gay and bisexual men who experienced victimization of homophobic bullying during childhood have self-identity disturbances in emerging adulthood [46]. Moreover, self-identity disturbance mediates the association between microaggression due to sexual orientation and mental health problems among young adult LGB individuals [47].

Although familial sexual stigma is specific to sexual orientation, it may make adolescent and young adult LGB individuals distrust their self-worth and disturb the establishment of self-identity. Moreover, family may provide the standards and references for adolescents to develop self-values [38]. Poor family support may reduce the individuals’ trust in and interaction with their families and interfere with the inheritance of family values, resulting in the disturbance of youth self-identity. Self-identity disturbance may also weaken the ability of adolescent and young adult LGB individuals to resist and cope with public stigma and increase the risk of internalized homonegativity. However, the mediating roles of self-identity disturbance in the associations of familial sexual stigma and family support with internalized homonegativity among LGB individuals have not been examined in previous research.

### Moderating effect of gender

Research has shown gender differences with respect to the number and forms of stigma-related stress [3]. Firstly, gay and bisexual men endure greater pressure to conform to a heteronormative gender role than lesbian and bisexual women [7, 48, 49]. Gay and bisexual men also experience higher rates of sexual victimization [50] and hate crimes [49] than lesbian and bisexual women. Furthermore, there are gender differences in the coming out experiences such as the awareness of same-sex attractions, first sexual experience, coming out in the gay and lesbian world, labeling oneself as gay or lesbian, coming out to friends, family, and co-workers, and coming out publicly of men and women related to conformity to and violation of sex-role expectations, as well as to political and legal issues [51]. For example, gay men first acted on their same-sex attractive feelings earlier and sooner than lesbian women [52]; women are less likely to label themselves 'lesbian' on the basis of a single same-sex involvement, while men experience the admission of such activity as implying the label 'gay' [53]; gay men perceive greater threatening feelings and resistance to be labelled as “homosexuality” compared with lesbian women [54]. Lesbian and bisexual women may also experience greater fluidity in their sexual orientation when compared to that of gay and bisexual men [55]. Finally, rumination was identified as a mediator of the association between sexual minority stressors and psychological distress among LGB individuals [56]. Research has also demonstrated depressive women are more likely to ruminate than depressive men [57], although whether the gender difference in rumination exists among LGB individuals experiencing sexual minority stressors is not ascertained. Such gender differences may lead to differences in the ways that internalized homonegativity is experienced by gay men and lesbian women [3, 58, 59]. However, whether gender may moderate the associations between familial sexual stigma, self-identity disturbance, and internalized homonegativity warrants further study.

### Aims of the present study

The present study has three aims. These were to examine the (i) associations of familial sexual stigma and perceived family support with internalized homonegativity among LGB young adults, (ii) mediating effect of self-identity disturbance in these associations, and (iii) moderating effect of gender in the associations of familial sexual stigma and perceived family support with internalized homonegativity and the mediation of self-identity disturbance. There were five hypotheses (Hs):

- H1: Familial sexual stigma is positively associated with internalized homonegativity among young adult LGB individuals.
- H2: Self-identity disturbance mediates the association between familial sexual stigma and internalized homonegativity among young adult LGB individuals.
- H3: Perceived family support is negatively associated with internalized homonegativity among young adult LGB individuals.
- H4: Self-identity disturbance mediates the association between perceived family support and internalized homonegativity among young adult LGB individuals.
- H5: Gender moderates the associations of familial sexual stigma and perceived family support with internalized homonegativity and the mediation of self-identity disturbance among young adult LGB individuals.

## Methods

### Participants and procedure

Participants were recruited by posting an online advertisement on social media, including *Facebook, Twitter*, and *LINE* (a direct messaging app), the *Bulletin Board System* (a popular application dedicated to the sharing or exchange of messages on a network), and the homepages of three health promotion and counseling centers for LGB individuals from August 2018 to July 2020. The inclusion criteria were individuals who identified their sexual orientation as being homosexual or bisexual, aged between 20 and 30 years, and living in Taiwan. Anyone who self-identified as a lesbian, gay, or bisexual and intended to participate in the present study could telephone the research assistants. The research assistants ensured the eligibility of potential participants for recruitment, explained the study aims and procedures to them, and scheduled the time for completing the study survey with them individually in the study room. Ten potential participants were screened out due to the ineligibility of age (younger than 20 years or older than 30 years). The research assistants interviewed the participants face-to-face in the study room to determine whether they had impaired cognition or any signs of alcohol and substance use that might interfere with understanding the study’s purpose and method or their ability to respond to the questions. If they had, they were excluded from the study. In accordance with the research plan, 500 male and 500 female LGB participants were recruited into the present study. Informed consent was obtained from all participants prior to the assessment. According to Kline [60], the number of the participants for structural equation modeling (SEM) used in the present study should be 200 or larger. Therefore, the sample size was large enough to examine the association among familial sexual stigma, family support, self-identity disturbance, and internalized homonegativity among gay and bisexual men and lesbian and bisexual women separately. Participants completed the study questionnaire individually in the study rooms and were assured that their responses would remain confidential. The study was approved by the Institutional Review Board of Kaohsiung Medical University Hospital (KMUHIRB-F(II)-20180018).

## Measures

### HIV and Homosexuality Related Stigma Scale (HHRS)

The 10-item Homosexuality subscale of the HHRS [61] was used to assess the stigma attitudes toward homosexuality that LGB individuals perceive from their families. The items (e.g., “My families unwillingly accept lesbian/gay individuals”) are rated on a four-point Likert type scale from 1 (strongly disagree) to 4 (strongly agree). A higher HHRS-Homosexuality score indicates a higher level of perceived stigma related to homosexuality from families. The HHRS-Homosexuality subscale has reported satisfactory reliability (Cronbach’s alpha 0.85) and psychometric properties in prior research [61]. The HHRS-Homosexuality subscale had satisfactory psychometric properties in the present sample (e.g., McDonald’s omega = 0.95 for male participants; and = 0.94 for female participants).

### Family Adaptation, Partnership, Growth, Affection, Resolve (APGAR) Index

The five-item traditional Chinese version [62] of the Family APGAR Index [63] was used to assess the five components of family support comprising adaptability, partnership, growth, affection, and resolve. The items (e.g., “I am satisfied with the help that I receive from my family when something is troubling me”) are rated on a four-point Likert type scale from 1 (never) to 4 (always). A higher total score indicates a higher level of perceived family support. The traditional Chinese version of the Family APGAR Index has reported acceptable discriminatory validity for social adaptability [62] and congruent validity with significant correlation with general health state [64]. Cronbach’s α of the FAI in the present study was 0.86.

### Self-Concept and Identity Measure (SCIM)

The traditional Chinese version [46] of the 27-item SCIM [41] was used to assess the level of current self-identity disturbance. The SCIM assesses three dimensions of self-identity disturbance comprising disturbed identity (e.g., “Sometimes I pick another person and try to be just like them, even when I’m alone”), unconsolidated identity (e.g., “When someone describes me, I am not sure if they are right or wrong”), and lack of identity (e.g., “I feel like a puzzle and the pieces don’t fit together”). Items are rated on a seven-point rating scale ranging from 1 (strongly disagree) to 7 (strongly agree). A higher total score indicates a higher tendency for self-identity disturbance. The traditional Chinese version of the SCIM has reported acceptable congruent validity with bullying victimization [41] and predictive validity for depression and suicidality one year later [65]. Cronbach’s α of the SCIM in the present study was 0.79.

### Measure of Internalized Sexual Stigma for Lesbians and Gay Men (MISS-LG)

The traditional Chinese version [49] of the 17-item MISS-LG [7] was used to assess the three dimensions of internalized homonegativity, including social discomfort sexuality and identity for LGB individuals. The items (e.g., “If it were possible, I would do anything to change my sexual orientation”) are rated on a five-point Likert type scale from 1 (strongly disagree) to 5 (strongly agree). The MISS-LG has two versions (one for lesbians and one for gay men) with the same factor structure. A higher total dimension score indicates a higher level of internalized homonegativity. The MISS-LG has reported satisfactory psychometric properties in prior research [7]. The results of Rasch and confirmatory factor analysis has confirmed the same three-factor structure of the traditional Chinese version of the MISS-LG (TC-MISS-LG) across gender used among young adult LGB individuals in Taiwan [66]. The TC-MISS-LG scores were significantly correlated with perceived social stigma toward sexual minority, supporting its concurrent validity. The McDonald’s omega of the three TC-MISS-LG dimensions ranged from 0.67 to 0.90, supporting its acceptable to excellent internal consistency [66].

### Demographic and sexual orientation factors

Information was collected concerning the participants’ gender, age, education level (high school or below vs. college or above), and sexual orientation (homosexual or bisexual).

### Data analysis

With the use of descriptive statistics, including means (and standard deviations) and frequencies (percentages), the participants’ demographics were analyzed. Then, zero-order correlations with the use of Pearson correlation coefficients were calculated to understand the correlations between age, familial sexual stigma, family support, self-identity disturbance, and internalized homonegativity. Moreover, Pearson correlations were calculated for the entire sample, as well as the male and female samples. Then, with the use of maximum likelihood estimator, SEM was performed to test the fit between data on the entire sample and the proposed model. Multigroup SEM without latent constructs on gender was then carried out to test the fit again. A good fit model should have the following fit indices satisfied: a nonsignificant χ^2^ test, comparative fit index (CFI) greater than 0.9, Tucker-Lewis index (TLI) greater than 0.9, root mean square error of approximation (RMSEA) less than 0.08, and standardized root mean square residual (SRMR) less than 0.08 [67]. For the multigroup SEM on sex, to see whether sex performed as a moderator was examined using χ^2^ difference tests. More specifically, a constrained path (i.e., forced path coefficients being equal between male and female samples) was compared with when it is freely estimated (i.e., the path coefficients were allowed to be different between male and female samples). The SEM was estimated using diagonally weighted least squares estimation. When a χ^2^ difference test suggests a significant difference between the constrained path and freely-estimated path, sex is evidenced to be a moderator for the path. The majority of the data analyses were done using the IBM SPSS 20.0 (IBM Corp., Armonk, NY); SEM and multigroup SEM were done using the lavaan package [68] utilizing R software.

## Results

The mean age of the participants was 24.6 years (SD = 3.0 years); the educational level was high (89.1% had a college or above degree). Over half of the participants self-identified their sexual orientation as homosexual (57.0%), with the rest of the participants self-identifying as bisexual (43.0%). Additional information regarding the participants is provided in Table 1, including their scores on the scales assessing familial sexual stigma, family support, self-identity disturbance, and internalized homonegativity. The absolute values of skewness and kurtosis of the continuous variables were less than 2, indicating normal distributions according to Kim [69].

**Table 1:** Characteristics of the sample (*N* = 1000)

	Mean (SD)	*n* (%)	Skewness	Kurtosis
Age (year)	24.6 (3.0)		0.121	-1.139
Sex				
Female		500 (50.0)		
Male		500 (50.0)		
Educational level				
High school or below		109 (10.9)		
College or above		891 (89.1)		
Sexual orientation				
Bisexual		430 (43.0)		
Homosexual		570 (57.0)		
Familial sexual stigma	26.6 (6.5)		-0.259	-0.181
Family support	13.6 (3.6)		-0.349	-0.326
Self-identity disturbance	86.8 (21.0)		0.183	-0.148
Internalized homonegativity	35.3 (11.5)		0.534	-0.264

Almost all the studied variables in the proposed model were significantly associated with each other (Table 2). More specifically, family support was negatively associated with familial sexual stigma (*r* = -0.21 to -0.27; *p* < 0.001), self-identity disturbance (*r* = -0.26 to -0.32; *p* < 0.001), and internalized homonegativity (*r* = -0.08 to -0.11; *p* = 0.001 to 0.07) for the entire sample, male sample, and female sample. Familial sexual stigma, self-identity disturbance, and internalized homonegativity were positively and significantly associated with each other (*r* = 0.15 to 0.38; *p* < 0.001) for the entire sample, male sample, and female sample. Multicollinearity among age, familial sexual stigma, family support, self-identity disturbance, and internalized homonegativity was examined by the condition index. The value of the condition index was 29.777; because it was less than 30, it indicated no problem of multicollinearity according to Hair [70].

**Table 2:** Zero-order correlation matrix between the studied variables

			*r* (*p*-value)		
**Entire sample**	1	2	3	4	5
1. Age	–				
2. Familial sexual stigma	0.06 (0.046)	–			
3. Family support	-0.05 (0.10)	-0.24 (< 0.001)	–		
4. Self-identity disturbance	-0.08 (0.02)	0.17 (< 0.001)	-0.30 (< 0.001)	–	
5. Internalized homonegativity	0.04 (0.24)	0.26 (< 0.001)	-0.11 (0.001)	0.37 (< 0.001)	–
**Male sample**					
1. Age	–				
2. Familial sexual stigma	0.08 (0.09)	–			
3. Family support	-0.03 (0.51)	-0.27 (< 0.001)	–		
4. Self-identity disturbance	-0.15 (0.001)	0.19 (< 0.001)	-0.26 (< 0.001)	–	
5. Internalized homonegativity	0.08 (0.09)	0.31 (< 0.001)	-0.08 (0.07)	0.38 (< 0.001)	–
**Female sample**					
1. Age	–				
2. Familial sexual stigma	0.04 (0.35)	–			
3. Family support	-0.07 (0.13)	-0.21 (< 0.001)	–		
4. Self-identity disturbance	-0.02 (0.62)	0.15 (0.001)	-0.32 (< 0.001)	–	
5. Internalized homonegativity	-0.04 (0.44)	0.21 (< 0.001)	-0.10 (0.03)	0.36 (< 0.001)	–

**Fig. 1:**
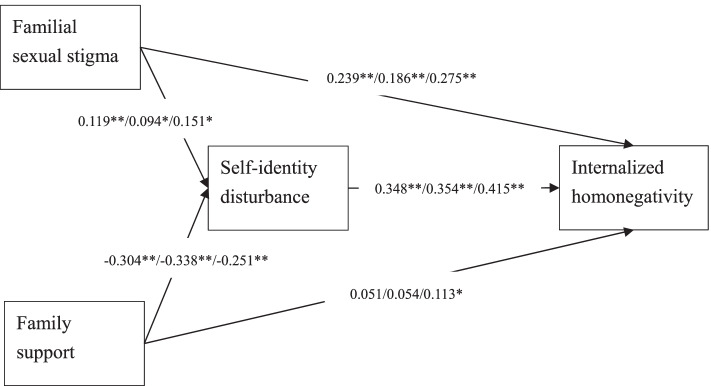
Structural equation model results with standardized regression coefficients for the proposed model. Age was controlled in the model. Standardized regression coefficients presented for the overall sample/ male sample/ female sample. *p < 0.05; **p < 0.001

The fit indices of the proposed model on the entire sample showed good fit, with the only exception being the significant χ^2^ test: χ^2^ = 140.39; df = 20; *p* < 0.001; CFI = 0.947; TLI = 0.913; RMSEA (90% CI) = 0.078 (0.066, 0.090); SRMR = 0.044 (Fig. 1). The fit indices of the proposed model using multigroup SEM also showed good fit, with the only exception being the significant χ^2^ test again: χ^2^ = 137.20; df = 40; *p* < 0.001; CFI = 0.955; TLI = 0.926; RMSEA (90% CI) = 0.070 (0.057, 0.083); SRMR = 0.038 (Fig. 1).

Regarding the path coefficients, they were all significant (*p* < 0.05) for the SEM on the entire sample and the multigroup SEM, except for the path between family support and internalized homonegativity. Familial sexual stigma was directly associated with increased internalized homonegativity, as well as being indirectly associated with increased internalized homonegativity via the mediation of self-identity disturbance. The results supported H1 and H2. The path coefficient between family support and internalized homonegativity was nonsignificant for the entire sample (standardized coefficient = 0.051; *p* = 0.15) and the male sample (standardized coefficient = 0.054; *p* = 0.28). In contrast, the path coefficient between family support and internalized homonegativity was significant for the female sample (standardized coefficient = 0.113; *p* = 0.02). The results did not support H3. Family support was indirectly associated with decreased internalized homonegativity via the mediation of low self-identity disturbance (supporting H4). χ^2^ difference tests further showed that all the path coefficients were not significantly different between male and female samples (χ^2^ = 0.02 to 0.52, df = 1; *p* = 0.47 to 0.88), except for the path between family support and internalized homonegativity (χ^2^ = 4.30, df = 1; *p* = 0.04). The results indicated that the moderating effect of gender existed only in the association between family support and internalized homonegativity. The results partially supported H5.

## Discussion

The findings of the present study showed that among LGB individuals, familial sexual stigma was directly associated with increased internalized homonegativity, as well as being indirectly associated with increased internalized homonegativity via the mediation of self-identity disturbance. Moreover, family support was indirectly associated with decreased internalized homonegativity via the mediation of low self-identity disturbance. The direct association between family support and internalized homonegativity was only found among lesbian and bisexual women but not among gay and bisexual men.

### Associations among familial sexual stigma, self-identity disturbance, and internalized homonegativity

Internalized homonegativity is the result of internalizing the public stigma perceived by LGB individuals as being due to their sexual orientation [2]. Family is the primary unit providing social values for the individuals [38], including acceptance or rejection of sexual minorities. Although peers, schools, and social media may also influence LGB individuals’ attitudes toward their own sexual orientation, familial sexual stigma may have a fundamental and longstanding influence and contribute the formation of internalized homonegativity among adolescent and young adult LGB individuals. The result confirmed the hypothesis made based on Bronfenbrenner’s socio-ecological theory. The present study’s findings also showed that self-identity disturbance mediated the association between familial sexual stigma and internalized homonegativity. The conflict between the sexual orientation and familial stigma due to being a sexual minority may make it hard for such individuals to integrate the values from various sources and develop an emaciated and inclusive self-identity. LGB individuals who have self-identity disturbance may fail to demonstrate stable beliefs, attitudes, and values [40, 71]. They may also tend to acquire the thoughts, feelings, and beliefs of others [39, 71]. Therefore, self-identity disturbance may increase the possibility for LGB individuals to agree, adopt and internalize the perceived sexuality-related public stigma. The result extended the minority stress theory [2, 3] by determining the mediation of self-identity disturbance in the association between familial sexual stigma and internalized homonegativity among LGB individuals.

### Associations among family support, self-identity disturbance, and internalized homonegativity

The present study’s findings showed that among LGB individuals, family support was negatively associated with self-identity disturbance and then was negatively associated with internalized homonegativity via the mediation of self-identity disturbance. Family support is one of the important factors protecting LGB individuals from mental health problems caused by sexuality-related public stigma [72]. Inadequate family support contributes to the development of suicidality and internalized homonegativity among young LGB individuals [73]. Moreover, according to the transactional model of developmental psychology [74], developmental changes occur as a result of continuous reciprocal interactions between an active organism and its active environmental context. Adolescents who perceive they have a warm and protective family climate have a high degree of mature identity [75]. Self-identity disturbance may further increase the risk of internalized homonegativity.

The direct association between family support and increased internalized homonegativity was only found among lesbian and bisexual women but not among gay and bisexual men. Research has demonstrated that females reported lower levels of family support in the process of growth compared with males [76–78]. It is possible that females are more sensitive to family interactions compared with males, as well as that traditional gender norms lead parents to more control and conflict with female than with male offspring [79]. Gender differences were also found in the association between perceived family support and behavioral health. For example, higher levels of perceived family social support were associated with lower odds of alcohol use and engagement in early sex in females but not in males [78]. Although the present study could not answer how gender moderated the association between family support and internalized homonegativity, the result suggests that intervention programs for enhancing family support to reduce internalized homonegativity should take gender into consideration. In addition to gender, religious affiliation has been found to be significantly associated with increased internalized homonegativity [80]. Further study is needed to examine the moderating effect of religious affiliation on the associations of familial sexual stigma and perceived family support with internalized homonegativity.

### Implications

Internalized homonegativity is an important treatment issue and is related to comfort in discussing various concerns central to sexual identity and expectations for level of personal commitment to the counseling process [59]. There have been several intervention programs developed to reduce internalized homonegativity among LGB individuals, mainly focusing on coping to sexual minority stress, disclosing sexual orientation, and enhancing mental health [81]. An online intervention designed to increase awareness of stereotypes, reflect on the sources of misconceptions, and increase self-affirmation demonstrated a statistically significant but small reduction in an explicit measure of internalized stigma [82]. However, another study based on the LGB-affirmative cognitive-behavioral therapy protocol did not exert a statistically significant effect on internalized homonegativity relative to a waitlist control [83]. Research has found that having a mindful nonjudging attitude toward one's inner experience is associated with less internalized sexual stigma among LGB individuals [84, 85], indicating that the effects of mindfulness-based interventions on reducing internalized homonegativity deserves further investigation.

There is no study proposing interventions addressing familial sexual stigma and their effects on reducing internalized homonegativity among LGB individuals. Based on the results of the present study, there is a need to develop intervention programs for reducing familial sexual stigma and it will be important to examine the effect of such interventions on internalized homonegativity among LGB individuals. Research based on identity status theory [86] has demonstrated that clarifying individuals’ identity through fostering exploration may enhance identity commitment [87] and helps individuals become more mature and competent during life transitions [88]). Moreover, intervention programs designed to enhance family support for LGB individuals should enrich relationships and communication between families and LGB individuals as well as families’ knowledge regarding sexual orientation [89]. However, whether these interventions can reduce internalized homonegativity among LGB individuals warrants further study.

### Limitations

There are some limitations in the present study. First, the cross-sectional study design limited the inferences concerning the temporal relationships between familial sexual stigma, self-identity disturbance, and internalized homonegativity. Second, the present sample comprised young adults (aged between 20 and 30 years) who were well-educated (nearly 90% of the participants had a college degree or above). Research has found that age was significantly associated with increased internalized homonegativity among LGB individuals [7, 90]. With respect to educational level, Berg et al. [91] found that internalized homonegativity was higher among gay and bisexual men with higher education, whereas Jacobs et al. [92] found that internalized homonegativity was higher among gay and bisexual men who had lower education levels. It is unclear whether the results of the present study could be generalized to the populations with other age ranges or with lower levels of education. Third, all the data collected in the present study were self-reported. Therefore, single-rater biases, recall biases, and social desirability biases cannot be fully controlled. Fourth, the present study asked for participants’ gender identities using the binary distinction of male and female but did not include the options of transgender, gender nonbinary, or genderqueer. Research has indicated that sexual and gender minority identities have intersectional impacts on health [93] and behaviors [94]. Both sexual and gender minority identities should be considered in public health practice [95]. Last, participants were recruited via social media. Although recruiting participants through social media such as *Facebook* is a promising research method to target the minority population and deliver the message to large numbers of participants quickly [96, 97], social media users may not be representative of the population. A review of a study that recruited participants through *Facebook* reported a bias in favor of women, young adults, and people with higher education and incomes [97].

## Conclusions

The present study demonstrated that familial sexual stigma was directly associated with increased internalized homonegativity, as well as being indirectly associated with increased internalized homonegativity via the mediation of self-identity disturbance among LGB individuals. Family support was indirectly associated with decreased internalized homonegativity via the mediation of low self-identity disturbance LGB individuals. Health professionals should evaluate stigma due to being a sexual minority and provide support for their children among the families of LGB individuals. Intervention programs to reduce familial sexual stigma and enhance family support for LGB individuals and their families are necessary. Self-identity disturbance should also be assessed among LGB individuals. Interventions to prevent internalized homonegativity among LGB individuals is necessary, especially for LGB individuals with familial sexual stigma, low family support, and self-identity disturbance.

## Data Availability

The data will be available upon reasonable request to the corresponding authors.
